# An International Investigation of the Prevalence of Negative Visitor Behaviour in the Zoo

**DOI:** 10.3390/ani13162661

**Published:** 2023-08-18

**Authors:** Courtney Collins, Yotam Barr, Sean McKeown, Juan Scheun, Claudia Tay, Ruth O’Riordan

**Affiliations:** 1 School of Biological, Earth and Environmental Sciences and the Environmental Research Institute, University College Cork, T23 N73K Cork, Ireland; r.ramsay@ucc.ie; 2 Safari Ramat Gan, Ramat Gan 5225300, Israel; yotambarr@gmail.com; 3 Fota Wildlife Park, T45 CD93 Carrigtwohill, Ireland; sean@fotawildlife.ie; 4 Nature Conservation Department, Tshwane University of Technology, Pretoria 0183, South Africa; scheunJ@tut.ac.za; 5 Mandai Wildlife Group, Singapore 729826, Singapore

**Keywords:** zoo visitors, negative behaviour, animal behaviour, visitor studies

## Abstract

**Simple Summary:**

Visitors may affect zoo animals as they view them. If visitors engage in negative behaviours such as feeding, banging or touching, this has the potential to harm the animals. This research was an international project involving four zoos that quantified the occurrence of negative visitor behaviour through observation. The research found that negative visitor behaviours were common and banging was the most frequently observed negative action. The type of animal, the zoo it was housed in and the number of visitors present all affected the frequency of negative visitor behaviours. Charismatic species were the most harassed animals in the study, while children were the most likely to engage in negative behaviour. Negative visitor actions occurred more frequently when animals were active and in close proximity to visitors. It is important for zoos to be aware of their visitors’ behaviour so that they can minimise negative actions which could harm animals.

**Abstract:**

Negative visitor behaviour is an understudied area of zoo research, even though negative actions can have serious implications for animal welfare. This research project investigated the prevalence of negative visitor behaviours at four different zoos. It included observations of visitors at seven different taxa exhibits and three different types of enclosures. A modified version of behaviour sampling was used to record visitor behaviour and the activity of the animals, while a negative binomial regression was conducted to test the significance of several predictor variables against the number of negative behaviours observed. Negative visitor behaviour was relatively common, occurring in 57% of observations. Banging was the most commonly observed negative action. Negative behaviours were influenced by zoo (*p* < 0.001), species (*p* < 0.001) and the number of visitors present (*p* < 0.001). The charismatic species were the most harassed animals included in the study, while children were the most likely to engage in negative behaviour. Negative visitor behaviours occurred more frequently when animals were active and in close proximity to visitors. It is imperative for zoos to understand visitors’ behaviour so that they can effectively communicate with their visitors to minimise negative actions and promote better animal welfare.

## 1. Introduction

Zoos by their very nature bring people and animals into close proximity [1]. In fact, visitors are a daily part of life in the zoo and are what differentiate zoos from other animal facilities such as farms, sanctuaries or laboratories [2]. There is no doubt that visitors arrive at zoos expecting to see animals [3], and research has confirmed that the public prefer to see easily visible, active, charismatic animals in naturalistic enclosures [4,5,6,7]. Indeed, positive zoo visitor experiences with animals can lead to favourable emotional experiences [8], enhanced education [9] and financial contributions [3], which may help advance the conservation movement [7,10].

Contradictorily, most zoos are financially dependent on visitors and must provide engaging interactions to attract the visiting public; however, the visitor experiences must not infringe on positive animal welfare [3,11]. Opponents of zoos argue that public amusement is not a sufficient reason for keeping animals in captivity, even if entertainment is a vital function of modern zoos in attracting visitors [12]. As a way of balancing visitor needs with animal welfare, many zoos offer animal–visitor interactive (AVI) experiences. AVIs are common worldwide, with petting being the most common activity, though regional differences amongst AVIs are reported [13]. While there is some evidence that these activities may be beneficial for both the animals [14] and the public participants [15], this has not been universally found [16]. Furthermore, many zoo goers do not avail of these experiences, for financial or other reasons, and visit zoos solely for entertainment and socialisation [10,17,18]. To ensure the welfare of captive animals, zoos must consider the effect of all members of the public on their animals.

It is now well understood that visitors have the ability to affect zoo-housed animals. Previous research has summarised that captive animals’ behaviour may be affected by the visiting public in three different ways [19]. First, visitors may be perceived as a positive experience by animals, with visitors providing an enriching atmosphere for animals [20]. Second, the animals may have habituated to visitors so that their presence has a neutral effect and no behavioural response is detected [21]. Third, visitors could be a negative stimulus for captive animals, leading to stress and potentially reduced welfare [22]. Of course, many different factors affect animals’ behavioural responses to visitors, including species evolutionary traits [1], individual animal differences or personality [23,24], previous experiences with humans [2], enclosure design including optional retreat space [25,26], visitor proximity [27], as well as the behaviours that the visitors are engaged in [28]. For example, active, loud, fast and unexpected visitor behaviours may be more disturbing to captive animals than quiet, passive visitor behaviours [1,29].

However, there is a significant lack of research investigating visitors’ specific actions, beyond noise [30], stay-time [6] and engagement or attentiveness [31], while viewing captive animals. Ross and Lukas [32] observe that zoo staff believe that visitors spend a large amount of time harassing animals (e.g., banging on glass), yet there has been almost no investigation into the prevalence of negative visitor behaviour in the zoo. In fact, Collins et al. [33] state that most evidence of negative visitor behaviour in the zoo comes from research which attempts to reduce it at specific enclosures through regulatory signage or physical barriers [21,27,34,35], but few studies consider the actual type and frequency of negative visitor actions. Tay et al. [35] have recently discovered that staff presence may be the most significant deterrent for negative visitor behaviour.

One of the first studies to comprehensively quantify negative visitor behaviour at an Irish zoo included 25 different animal enclosures at one zoo [33]. The authors found that visitor banging was the most prevalent negative behaviour, while Humboldt penguins, lion-tailed macaques and Sumatran tigers were amongst the most harassed species. Negative visitor behaviour was associated with increased visitor numbers and traditional-style viewing areas. Higher visitor numbers and more negative visitor behaviour was also associated with more active animal behaviours. While it is likely that much of the negative visitor behaviour observed in the zoo is an attempt to connect with animals rather than cause harm [8], this behaviour should not be ignored or underestimated because it has the potential to reduce animal welfare and have serious consequences for visitors [1,33].

Another area of zoo research where there is an exceptional lack of knowledge is international differences in research findings and collaborative research between zoos in different locations. Cultural differences in how visitors view animals may have a profound impact in the area of animal welfare and visitor effect studies [36]. For example, Tishler et al. [37] investigated how different cultural groups in Jerusalem, Israel perceived the zoo. The authors reported that regardless of background, most visitors had positive feelings toward the zoo and regarded it as an educational institution; however, there were religious and cultural differences in areas such as favourite animal and interpretation of zoo messaging. In a review of animals in environmental education research, one author states that there is a lack of cultural diversity amongst research subjects and that most studies reviewed included ‘white, middle-class Westerns’ as visitors and there is ‘an urgent need for more intercultural and international research’ [38] (p. 70).

As a complex picture of the intricacies of human–animal relationships and visitor effects in the zoo begins to emerge, it is apparent that currently there are more questions than answers. However, in the interest of animal welfare, researchers must endeavour to investigate this multifaceted area. One of the most understudied topics in this field is onsite visitor viewing behaviour and cultural diversity in zoo research. The objective of this international research project is to investigate the prevalence of negative visitor viewing behaviour in zoos at four different worldwide locations. This includes several variables such as species, enclosure types and visitor demographics. The current research builds on the results of Collins et al. (2023), which investigated visitor behaviour at one zoo in Ireland, and together, these two studies form a foundation for researchers to begin to understand negative visitor behaviour, which could ultimately affect animal welfare.

## 2. Materials and Methods

### 2.1. Study Sites and Species

The research was conducted at four institutions:

1. Fota Wildlife Park (FWP), Carrigtwohill, Ireland
2. Mandai Wildlife Group (MWG), Singapore, Singapore
3. The National Zoological Gardens (NZG) of South Africa, Pretoria, South Africa
4. Safari Ramat Gan (SRG), Ramat Gan, Israel

Zoos were recruited for participation in the project through personal connections, word of mouth and email appeal. The research was due to begin in early 2020 with over 14 participating institutions; however, the COVID-19 pandemic severely affected the schedule of the research as well as the number of institutions that were able to participate. Given the length and severity of the pandemic and its varying effect on different regions and the closing of many zoological institutions, the remaining four participants collected data when it was feasible to do so based on zoo openings in their country (Table 1A–D). Thus, the data collection took place between July 2020 and August 2021. A variety of animal taxa were included in the study. Given the inherent differences in zoos’ collections, it was not possible to standardise the species included in the research at each zoo. Inclusion was decided based on animals that are common in zoos worldwide, popular with visitors, particularly charismatic such as the giant panda [39], or of specific conservation importance to a zoo, such as the cheetah at FWP. It was not feasible to specify which species were included in the research. Thus, the decision was generally made at class or family level. Because of known differences in how visitors respond to different animals [33] and how different animals respond to visitors [11,19], it was considered important to include a broad range of animals in this research. For example, by differentiating between the apes and other primates (small-bodied), it may be possible to disentangle where differences occur. Where possible, the zoos participating in this research included observation at the following animal exhibits:

1. Felid—big cat (e.g., *Panthera* spp.), not present at SRG
2. Penguins (e.g., *Pygoscelis* spp., *Spheniscus* spp.)
3. Meerkats (*Suricata suricatta*)
4. Small-bodied primates (e.g., Lermuridae; Cebidae; Cercopithecidae)
5. Apes (e.g., Hylobatidae; Hominidae)
6. Reptiles (e.g., Squamata; Testudines, Crocodilia), not available at FWP due to COVID restrictions
7. A particularly charismatic species/exhibit (giant panda *Ailuropoda melanoleuca*, cheetah *Acinonyx jubatus*, a petting zoo), not present at NZG

Exhibits and species varied at each zoo (Table 1 and Table 2). Data were generally collected in summertime during busy visitor periods at each location. Therefore, within each institution, there was little variation in the climate, and data were not collected if the weather was inclement, with the exception of Singapore, where rain is common and difficult to avoid. Once an institution agreed to participate in the project, they completed a collaborative research agreement form. Each participating partner was responsible for receiving ethical approval from their own institution and complying with their ethical guidelines, which varied between institutions. Overall project approval was given by Fota Wildlife Park’s Research Ethics Committee.

### 2.2. Procedure

Before the study began, participating zoos were issued strict procedural guidelines for data collection to maintain consistency and reliability between institutions. Additionally, the primary researcher at each institution met on-line with the coordinating researcher to discuss project logistics and clarify terms and definitions. Within each zoo, data were collected by only one observer. However, at Safari Ramat Gan, this was not feasible, and several observers were involved in data collection. Because staff had relocated, it was not possible to conduct traditional inter-observer reliability testing, where each researcher would observe and record the same behaviours. Therefore, to show reliability, the Kruskal–Wallis test was used on all of the data from SRG to test for consistency between observers on the total number of negative behaviours observed.

Observations took place at pre-determined ‘busy’ visitor times in each institution. For example, at Fota Wildlife Park, all of the observations took place between May–July of 2020, from 12 p.m. to 2 p.m. Researchers were instructed to wear plain clothes and unobtrusively observe visitors at the main viewing area of an exhibit, which was predetermined by the researcher as the area where visitors and animals were most likely to be seen. Since some enclosures may have multiple areas, including both indoor and outdoor areas, pre-determining observation points made data collection more consistent [40]. At times, researchers moved slightly to observe a visitor, but all observations occurred from approximately the same vantage point [33].

Only one observation session occurred per enclosure per day. Each exhibit included in the study was observed for a 30 min period and was replicated 10 times, yielding 300 min of observation per enclosure. During the 30 min period, using behaviour sampling, the total number of negative behaviours displayed by any visitor were counted (Table 1) [41]. These were identified as behaviours that are not compliant with the rules of the institution (Table 2) [28]. The researcher also observed what the negative behaviour was and the approximate age, gender and group composition of each person who engaged in a negative behaviour, as well as the animals’ location, activity level and proximity to visitors (Table 2). No personal or identifying visitor information was collected.

**Table 2:** (**A**–**C**) Test variables with response category definitions described, and how these data were used in the analysis: (**A**) Negative visitor behaviour and enclosure details, (**B**) visitor demographics, and (**C**) animal activity.

Variables Observed	Response Categories and Definitions	Data Analysis
**A. Negative behaviours and enclosure details**		
Negative visitor behaviour	Banging—striking or knocking on a glass window, wall or other structure in or near the enclosure with a hand or other object. If a visitor bangs several times in quick succession, this is one banging incident. If the bangs are separated by more than five seconds, these are individual bangs. This is an intentional action, though it might not be delivered in an aggressive manner. Shouting—a vocalisation that is louder than a normal talking voice and is directed at one or more of the animals in the enclosure. Feeding—offering food, throwing food or attempting to feed any food or non-food item to an animal. Food does not have to be accepted or consumed by the animal to be counted. Touching—physical contact or an attempt to make contact in a non-aggressive way; reaching towards an animal or through the enclosure with a hand, finger, stick or another object, such as a leaf, stick, toy, bottle, etc. Throwing—throwing or attempting to throw any object (not food—see No. 3 above) at an animal or into its enclosure. Chasing—running after an animal or charging an enclosure Climbing barrier/entering enclosure—fully entering an enclosure; or, if the visitor has more than two limbs and/or more than 50% of their body over a barrier, including leaning into or over a barrier with more than 50% of the body; or putting an object, such as a buggy, over the barrier or fence. Other—any behaviour that does not fit in the above categories; e.g., flash photography; kicking, spitting, etc.	Dependent variable model I
Enclosure type	Traditional—the animals’ movements are restricted to the enclosure; there are significant visible barriers between the animal and the public. There are few if any ‘natural materials’ or features present in the enclosure. This is unlikely to be a mixed-species exhibit. This type of enclosure has also been described as a first- or second-generation exhibit [42]. Naturalistic—the animals’ movements are restricted to the enclosure area. Significant barriers may separate the animals from the public, but these are often concealed. The enclosure has a natural appearance, perhaps resembling the animals’ native habitat, including grass and/or rocks and/or trees and/or water features present. This could be a mixed-species exhibit and could be described as an immersive exhibit or third-generation exhibit [42]. However, for the purposes of this study, visitors cannot enter the exhibit or come into close contact with the animals. Walk through—the animals’ movements are restricted to the exhibit area. This type of exhibit is similar to naturalistic enclosures but will have a path or walkway going through it. The visitors essentially view the animals from inside the enclosure, which allows visitors to see the animals in close proximity, but there are still restrictive barriers in place, perhaps fencing along the walkway. This may also be referred to as an immersive exhibit; however, the visitors enter into an enclosure and walk through it to see the animals [43]. Semi-free range—there are some restrictive barriers in place to protect visitors and animals, but the animal may be able to roam freely at certain times of day or under certain conditions. Or, the animal may be able to roam freely in a restrictive area that visitors can enter. This could be similar to a walk-through exhibit, but the animals’ movements within the enclosure are completely unrestricted. The animal and visitor can come into direct contact. These are referred to by Mun et al. [44] as free-ranging exhibits. To differentiate from those that are truly free-ranging, where the animals could actually leave the zoo [45], they are called semi-free-ranging for the purposes of this study. The current study did not include any truly free-range exhibits.	Independent variable model I
Viewing proximity	Access unrestricted; animals and visitors can come into direct contact; generally a semi-free-range enclosure. Visitors can come within 1 m of animals; some type of barrier is in place (e.g., bushes, stones, a glass wall, etc.), but visitors may still be able to touch or feed the animals. Viewing distance is greater than 1 m; a larger barrier is in place (e.g., a moat); the animals may be located on an island or are viewed from a viewing platform that is above or below the enclosure. This would generally mean that visitors could not actually touch the animals.	Independent variable model I
Staff present	No—staff are not present at the enclosure Yes—staff are present at the enclosure	Independent variable model I
Signage present	No—signage, outlining visitor rules, is not present at the enclosure Yes—signage, outlining visitor rules, is present at the enclosure	Independent variable model I
Enrichment present	No—enrichment is not present at the enclosure Yes—enrichment is present at the enclosure	Independent variable model I
**B. Visitor demographics** **† and comments of visitors displaying negative behaviour**		
Age	Adult—18 years or more Child—under 18 years	Variable used in correlation matrix
Gender	Male Female	
Group composition	Alone—not with a group School group—a group of children perhaps with adult supervisors Family group—a group of adults and children Other social group—an adult group or a few children without an adult	
Conversational comments	Management—directional, e.g., ‘Look!’, ‘Watch this’, ‘Over there/here’. Anthropocentric—human oriented, e.g., ‘I’ll make it/her/him move’. Discussion of engaging in a negative behaviour—e.g., ‘Let’s feed them’, ‘Give them this’, ‘I’ll touch one’. Animal centred—anything about the animal’s description or behaviour, e.g., ‘They’re big’, ‘It’s eating’. Emotive—‘I love them’, ‘He’s cute’, etc. Science/Conservation related—‘They’re endangered’ etc. Non-zoo—anything not having to do with the zoo, ‘When’s lunch’, etc. Other—any comment that does not fit these categories. No comment was heard.	
**C. Animal activity**		
Visibility	Poor—none of the animals are clearly visible Good—at least one animal is clearly visible	
Proximity	Far—all of the animals are at least 3 m away from the visitor Near—at least one animal is within 3 m of the visitor	
Activity	Inactive—the animal(s) are generally inactive or asleep Active—at least one animal is engaged in animated active behaviour	

† Based on the best estimation of the researcher.

The petting zoo presented a unique challenge to standardise data collection, since limited interactions with the animals were allowed. Therefore, for the petting zoo, negative behaviours were considered behaviours that went against the rules of the exhibit. For example, feeding, shouting, climbing barriers or on animals and aggressive handling were not permitted. For the purpose of this research, these were counted as negative behaviours (Table 1D).

Additionally, in an effort to begin to uncover why visitors engage in negative behaviour, the Tunnicliffe Conversation Observation Record (TCOR) [46,47] was used. This included a list of pre-designated conversation categories based on the TCOR but adapted to fit the current study (Table 2). Observers noted any conversational comments that visitors made while engaging in negative behaviour. During the 30 min observation period, instantaneous scan sampling, with a 5 min interval, was also be used to count the number of visitors present at the enclosure during the sample interval [48]. This was averaged to give the mean number of visitors present during the 30 min observation session (Table 1).

Finally, for each species involved in the study, the main staff member responsible for the animal was asked by the researcher to complete a one-question survey on their perception of visitor behaviour towards the animal. This was conducted once the data collection was complete (Table 1). Staff were asked, on a scale of 1 to 5, how much ‘negative’ visitor behaviour does this animal enclosure receive? Response options were as follows:

1. Never; visitors do not engage in any behaviours that are against the rules of the institution. I almost never see negative behaviour at this enclosure.
2. Rarely; visitors generally do not engage in negative behaviour, but there may be the occasional person that engages in negative behaviour. I see negative behaviour once or maybe twice a week.
3. Sometimes; sometimes visitors engage in negative behaviour at this enclosure. I see negative behaviour at the enclosure several times a week
4. Often; visitors regularly engage in negative behaviour at this enclosure. I see it many times a week and once or twice a day
5. Always; visitors are constantly engaging in negative behaviour at this enclosure. It appears to happen several times every day of the week.

### 2.3. Data Analysis

To begin, the Kruskal–Wallis test was used to determine if there was a significant difference between the individual observers at one of the institutions. It was found that there was no difference between the number of negative behaviours observed by each researcher (χ^2^ (6) = 8.90. *p* = 0.18). Therefore, it was decided to include observations from all of the observers at Safari Ramat Gan in the subsequent analysis.

First, data are presented using descriptive statistics. The data violated the assumption of normality and equi-dispersion, so a negative binomial regression was conducted [49] to test the significance of seven predictor variables (zoo, species, enclosure type, enrichment presence, staff presence, signage presence and viewing proximity), which were based on the categories originally provided to the participating zoos, against the response variable (the total number of all negative behaviours observed per observation session) (Table 1 and Table 2). Although staff presence [35] and enclosure type may have an impact on the number of negative behaviours observed, these variables were removed from the model because of significant imbalances in the dataset. Additionally, this affected the addition of the intended interaction terms in the model (zoo*species, species*enclosure type and zoo*enclosure type). It was still possible to include the interaction term zoo*species since most taxa were represented at each zoo. Then, a backwards stepwise approach was used to remove variables with the largest *p*-values (>0.05) from the model, until a simplified model that best fit the data was achieved (Table 3). Where a significant difference occurred, post hoc testing using the Tukey test was applied. All assumptions of the model were met.

Next, a Kruskal–Wallis test was conducted to test for differences in negative visitor behaviour rates between the four enclosure types. This was done in addition to the original model due to the elimination of enclosure type, despite its expected significance. Because of an uneven distribution of the types of enclosures present in this study, these data were also tested using enclosure type as binomial variable (either traditional or non-traditional) with the Mann–Whitney *U* test.

When a negative behaviour occurred, in order to show relationships between the different variables, a contingency table was created (Table 5). However, visitor group type and animal visibility were discounted since 75% of groups were families and at least one animal was visible in 99% of observations. Therefore, visitor age, visitor gender, animal proximity, animal activity level and zoo were tested against the different types of negative visitor behaviour that were observed. Due to an expected frequency of less than five, the Fisher’s exact test was used to analyse gender, proximity, and activity, while age and zoo were tested using Chi-square since they met the assumptions of the test.

Finally, to test for an association between staff’s perception of visitor behaviour towards specific species and visitors’ actual behaviour towards those species, a Spearman rank order correlation test was conducted. First, the average number of negative incidences was calculated for each study species at each zoo, then this number was compared to staff response on the survey.

Bonferroni corrections were applied when multiple comparisons occurred. Data analysis was conducted using SPSS version 28 and Microsoft Excel 365 [50]. The accepted alpha level for these analyses was *p* < 0.05 unless otherwise stated.

## 3. Results

### 3.1. Descriptive Statistics

A total of 300 min of observation occurred at each enclosure in the study, except for SRG, where two species were only observed for 270 min each. This yielded a total observation time of 7740 min. At all institutions, the number of visitors present at the enclosures ranged from 0 to 259 (x = 54 ± 3.15 SE). In total, 412 negative behaviours were observed (Table 1 for the total number of negative incidences observed at individual exhibits, Table 5 for negative behaviours represented by tested categories). This ranged from 0 to 10 (x = 1.60 ± 0.11 SE) per observation session at each enclosure. A negative behaviour occurred in approximately 57% of observations. The most prevalent negative behaviour was banging (42%), followed by shouting (27.2%), climbing fence/entering enclosure (13.3%), touching (5.6%), feeding (5.3%), throwing (3.6%), other (1.9%), and chasing/kicking (1%). Children (73.5%) were more likely than adults (26.5%), and male visitors (64.8%) were more likely than female visitors (35.2%), to engage in negative behaviour. Family groups (75%) were most likely to engage in negative behaviour, followed by other social groups (14.3%), schools (7.5%) and solo visitors (3.2%). When a negative behaviour occurred, comments by the visitor were observed to occur 50% of the time. When a comment occurred, the most frequent remark was one of management (26.7%), followed by an animal centred comment (10.7%). All other conversational remarks (Table 2) were below 5%, with conservation comments the most infrequent at 0.2%.

### 3.2. Model

**Figure 1:**
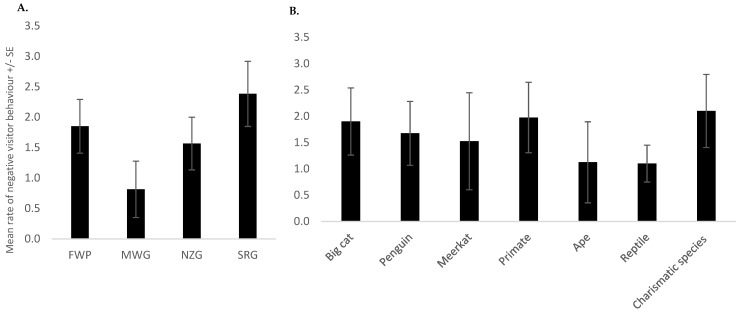
(**A**,**B**) Significant predictors, (**A**) the four zoos and (**B**) each species’ exhibit, of the mean number of negative behaviours observed per observation session +/− the standard error of the mean (SE). I See Table 4 for differentiating between zoos that did not have the species versus zero negative behaviours observed.

**Figure 2:**
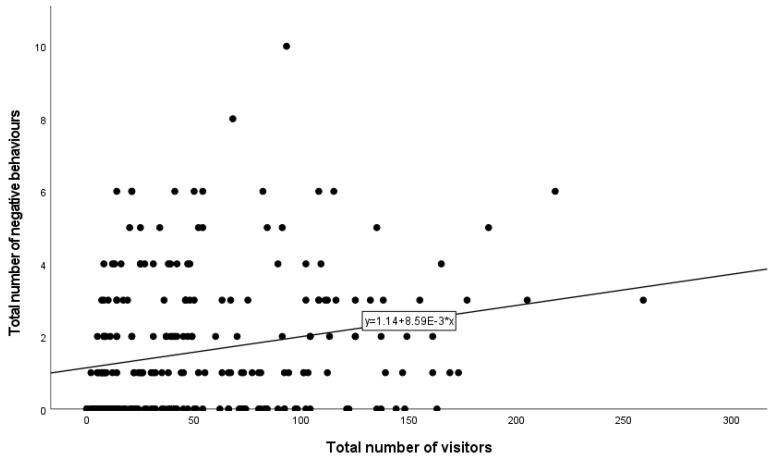
Significant predictor, the total number of visitors present per observation session at all zoos included in this research, versus the total number of negative behaviours observed per observation session.

Results from the negative binomial regression showed that zoo (*p* < 0.001) (Figure 1A) and species (*p* < 0.001) (Figure 1B) remained in the model as significant predictors of the number of negative behaviours observed (Table 3). The highest rate of negative behaviour occurred at SRG, whereas the lowest occurred at MWG (Figure 1A). Tukey’s test revealed statistically significant differences between FWP and MWG (*p* = 0.005) and between MWG and SRG (*p* < 0.001). The highest rate of negative behaviour occurred at the charismatic species exhibits, followed by the primates and big cats (Figure 1B). However, after the Bonferroni correction was applied, the Tukey test did not reveal any significant differences between species. Additionally, there was a statistically significant interaction between zoo and species (*p* < 0.001) (Table 3 and Table 4). The number of visitors present (*p* < 0.001) was also a statistically significant predictor of negative behaviour (Figure 2 and Table 3). As the number of visitors increased, the number of negative visitor behaviours also increased (Figure 2).

The Kruskal–Wallis test did not reveal any difference in observed negative behaviour between the four enclosure types (χ^2^ (3) = 5.381; *p* = 0.146). When these data were tested as a binomial variable, the Mann–Whitney *U* test did not detect any difference in negative behaviour observed at either enclosure type (U = 7450.50; *p* = 0.487).

### 3.3. Correlations

**Figure 3:**
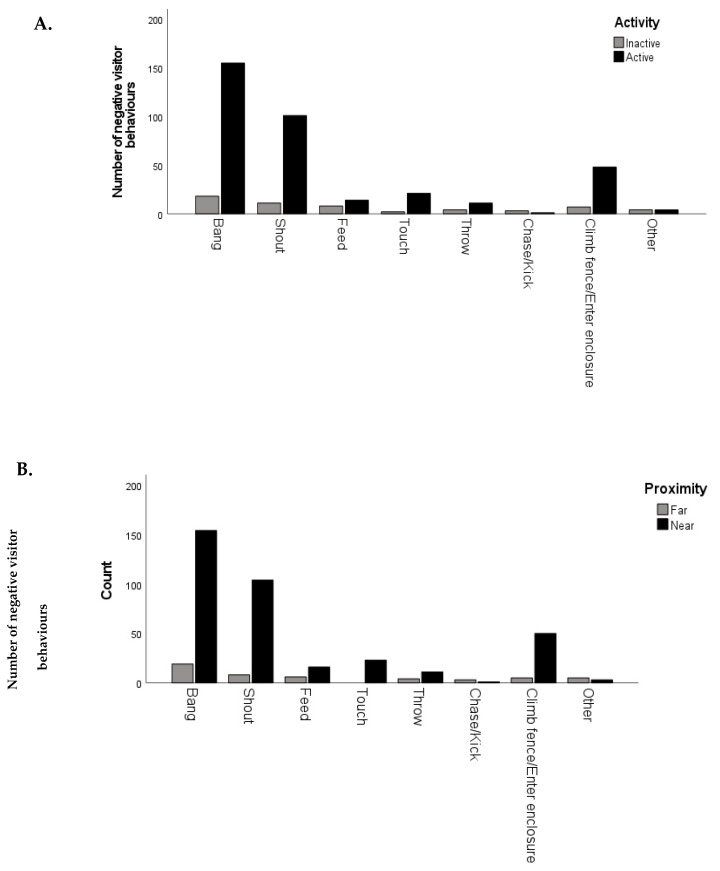
(**A**,**B**). The total number of negative behaviours observed when animals were (**A**) inactive or active or (**B**) far or near for each category of negative behaviour.

Tests of association showed a statistically significant and strong association of visitor age, animal proximity, activity level and zoo with the negative visitor behaviours observed (Table 5 and Figure 3A,B). The Spearman rank correlation test was conducted to test for a relationship between the average number of negative visitor actions per observation session and staff’s perception of how ‘harassed’ the animal was by visitors. A weak, positive correlation was detected, but this was not found to be statistically significant (r_s_ (24) = 0.343, *p* = 0.086).

## 4. Discussion

The findings from this study represent the first international investigation into the prevalence of negative visitor behaviour in the zoo. Results indicate that a negative behaviour occurred in 57% of observations, suggesting this is an area that warrants further investigation. A previous study at an Irish zoo reported a negative visitor behaviour in 18% of observations [33]. This difference could be due to variations between region or, more likely, between species included in the study. Similarly to Collins et al. [33], banging was found to be the most prevalent negative visitor behaviour in the current study, followed by shouting and crossing barriers, though this varied between zoos. These two noise-producing activities could be concerning since some captive animals are known to react negatively to noise [26,30]. Crossing barriers could have serious implications for both visitors and animals, and zoos should be made aware of these detrimental actions immediately. Tactile actions such as touching and feeding were not commonly observed during this research. However, it is important to consider that not all negative actions (e.g., feeding) were possible at every enclosure. Future research should tease this out to report which behaviours are most likely to occur at which enclosure. As found in previous research, children, men and family groups were the most likely to engage in unwanted behaviours [33].

In an effort to uncover why visitors engage in negative behaviour, any comments made by visitors behaving negatively were observed. Interestingly, many visitors did not say anything when acting undesirably. When they did say something, it was most often a management comment such as ‘watch this’. Conservation comments were the most infrequent, which coincides with what other authors have reported about a general lack of science and conservation dialogue at the zoo [17,52]. Though the current study was a limited attempt to discover the motivation behind visitors’ negative actions, the prevalence of management comments such as ‘watch this’ over other types of comments suggests visitors could be using forced animal interactions as a shared emotional experience to enhance their social experience during the visit [33,53]. The importance of providing visitors with shared emotional experiences with animals in the zoo is emerging as an important aspect of a zoo visit [53].

Zoo, species and number of visitors were found to be significant predictors of negative visitor behaviour. Of the four zoos, the highest rate of negative behaviour occurred at Safari Ramat Gan in Israel, while the lowest occurred in Mandai Wildlife Reserves, Singapore. Although the specific sociocultural demographics of visitors at these zoos were not collected, these data suggest that significant differences in negative behaviour occurred between these international locations. This supports limited previous research which reported that cultural differences affect how visitors view animals and interpret zoo messaging [36,37]. However, it is difficult to make further inferences from these data. There could be many other influences besides location, such as the zoo environment [6], that affected this result. However, since enclosure design was not found to be significant, it is likely that some aspect of the zoo’s location does affect behaviour. Further, in-depth research is needed in this sensitive area to ascertain if certain cultural, religious or societal differences contribute to visitors’ behaviour. Understanding how different cultures view captive animals could lead to important welfare findings [36] and is essential for zoos when considering how they communicate with their visitors.

Zoo visitor attendance is correlated to animal body mass, which is in turn linked to in situ conservation effort [54]. Furthermore, research has shown that species type affects visitor interest and ‘stay-time’ at exhibits and even affects the development of conservation behaviours [6,7,55]. If zoo visitors prefer certain animals to others, it is reasonable to expect different behaviour at different enclosures, as visitors either attempt to interact with their favourite animal or perhaps treat it with greater respect [33]. Nash [56] suggests that visitors frequently bang on the glass of reptile enclosures in order to provoke a movement or behaviour from animals perceived to be generally inactive. However, the results found here do not support that. While negative incidences did occur at reptile enclosures, they were low compared to other species. The most negative behaviour occurred at the enclosures with charismatic species. Indeed, the highest incidence of negative behaviour occurred at the giant panda exhibit, which was only present in MWG (Table 4), followed by the big cats and primates (across the four locations). These findings coincide with previous research which shows that visitors are drawn to charismatic megafauna, that is, large exotic animals, usually mammals [57,58]. Visitors may form emotional connections with these animals because they can empathise with them by seeing similarities to their own physical characteristics or behaviour [59,60]. Connections with zoo animals can lead to conservation caring, increased donations and the development of pro-conservation action, which is a positive result of visiting a zoo [57,60].

However, conflict can occur if visitors attempt to force interactions with their favourite animal through negative actions like touching, feeding or banging the glass. Across the four zoos included here, the primates and big cats consistently attracted the most negative behaviour. It is likely that visitors are seeking connections with these animals rather than attempting to harm them [8,33]. Interestingly, the apes received less negative attention than the smaller bodied primates. The difference here could be due to slight differences in enclosure design, which may have made physical interactions with apes more difficult; for example, an island or a naturalistic setting could have made negative actions like banging unfeasible. The petting zoo at SRG (goats and sheep) also attracted a large number of negative behaviours. Although visitors were allowed limited tactile contact, breaching barriers in restricted areas and illicit feeding attempts were frequently observed. A previous study of visitor effects on petting-zoo animals found almost no behavioural response from goats to visitors, and the authors concluded that their welfare was not negatively affected by visitors [61]. However, species differences did occur, and even if behavioural changes did not occur, unregulated feeding and breaching barriers could have negative physical consequences for visitors and animals. Although MWG reported the least negative behaviour of the study, it housed the most harassed species of the study—the giant panda. Endemism, limited geographic range, conservation threat level and cultural importance may all influence giant pandas’ popularity [60,62]. Again, it is likely that pandas’ charisma led to visitors attempting to force connections with them. In fact, the giant panda is one of the 20 most charismatic animals as rated by the Western public [55]. However, Tishler et al. [37] report cultural differences in animal preferences. While it is evident that giant pandas are popular in zoos, one study reports the public in Thailand protesting over zoos diverting funds to pandas over their native elephants [63]. Unfortunately, the giant pandas were only present in one of the zoos included in this research, so it is not possible to make comparisons between different areas, but this should certainly be an area for future research.

Interactions between the species and location also occurred. For example, the meerkat and the apes at Safari Ramat Gan received a very high level of negative behaviour compared to similar species at the other three zoos. The penguins at Fota received more negative attention than at the other locations, which supports previous research on this penguin enclosure [33]. It seems plausible that this is because of enclosure differences, yet the results found here did not indicate that negative behaviour was affected by enclosure type. This is a surprising result given that previous research has found that visitors behave differently at varying enclosure types [44,64,65]. Although Chiew et al. [65] summarise that there are many differences that can occur at zoos other than enclosure type, such as zoo membership, resident or tourist, pet ownership and level of education, it is likely that this type of visitor demographic, which was not evaluated in the current study, affected these results.

There was a strong correlation between negative behaviour and visitor numbers. This is similar to the findings reported by Mun et al. [44] and Collins et al. [33] that negative visitor behaviours increased with increasing visitor numbers. People engage in unwanted behaviours for a multitude of reasons, yet few visitor-related zoological studies are informed by psychological models such as the theory of planned behaviour [66]. Ajzen [66,67] described that behaviour is a result of three categories of salient beliefs: behavioural (beliefs and attitudes relating to the consequences of a behaviour), normative (beliefs about social pressure to engage in a behaviour) and control beliefs (beliefs about the ability to perform or control a behaviour). All of these could be important contributors to zoo visitor behaviour, but since many visitors see a zoo excursion as a social experience [17], normative beliefs may be particularly relevant. Normative beliefs or social norms imply that people should behave in a certain way but are receptive to the behaviour of others [68]. This can also be described as behaviour that is inherently understood by a group and is observed by the majority of people [69]. For example, there can be social pressure to behave in a certain way or not. Though not explicitly tested here, we suggest that increased group size leads to more negative behaviour because once one visitor engaged in a negative action, others may believe it is acceptable to duplicate the action. In short, negative behaviour could be transmittable. Or, visitors may believe that in a larger group, they are more likely to get away with a negative action. Interestingly, social norms are also culturally dependent [69], which would explain the differences in behaviour observed in the four zoos. This is an area that requires more in-depth research, but future zoo visitor studies should continue to consider the influence of culture and psychology when evaluating their visitors’ behaviour.

Age, but not gender, was found to be statistically significant in effecting the frequency of observed negative behaviour. More children than adults engaged in negative behaviours, except feeding, and it was observed by the researcher that often the adults in the group appeared to carry and control the food. Previous research has also found that children are more likely than adults to engage in negative behaviour [32,33]. However, it is also important to consider that children make up a large percentage of the visitors. It was not possible to quantify the percentage of children in attendance at each zoo in this study, but future research should investigate this further. Although children were the main perpetrators of negative behaviour, they are also susceptible to receiving the educational messages of zoos [70]. Furthermore, it is possible to reduce negative behaviour from children as they view animals in the zoo by implementing an educational intervention [28]. Previous research on gender differences in zoo visitors is minimal and reports mixed results. Women view animals with more emotion than men [59], which may explain why there was an observed trend that fewer women than men engaged in negative activities.

A limited investigation into actual number of negative incidences versus staff perception of negative incidences revealed a weak correlation, though this was not statistically significant. Ross and Lukas [32] also reported that staff complain that apes are commonly harassed by zoo visitors, though in reality visitors spent less than 0.03% of time engaged in undesirable behaviours. Still, 22% of visitors did engage in a negative behaviour [32], and it may be the frequency and not the duration of the behaviours that is disturbing. Zoo staff likely spend more time than anyone informally observing animal–visitor interactions, and they may also be a deterrent for negative visitor behaviour [3,35]. Thus, their perception of the treatment of zoo animals should be closely monitored and acted on if necessary.

A detailed investigation of animal behaviour was out of the scope of the current study. However, previous research has shown that animals’ behaviour can be negatively affected by zoo visitors [19] and that loud, fast and unexpected actions could be the most disturbing to captive animals [1]. Animal activity level and proximity to visitors have previously been used as indicators of animal response to visitors [27,44] so were considered a suitable proxy in the absence of analysing a full behavioural repertoire of animal behaviour. Visitors are drawn to and like to see active animals [4,10]. The results found here show that visitors are more likely to engage in negative actions when the animals are active, suggesting that the negative actions are visitors’ attempts to connect with the animals. This is further supported by the finding that visitors were more likely to bang or shout if the animals were closer to the visitors, presumably in an attempt to gain the animals’ attention. Myers et al. [59] found visitors had a stronger sense of connection with an animal if it paid attention to them. These results concur with Mun et al. [44], who report that animal–visitor interactions are more likely to occur when the animals are in close proximity to visitors. Although their study focused solely on primates, who may be the most likely to approach visitors, particularly if there is food involved [44,71]. One previous study found that little penguins reacted to visitors in close proximity with a fear response [27]. The paradox is that as visitor numbers increase and more negative behaviours occur, the more likely it is that the animals will retreat and become inactive or not visible [1,33].

Zoos must balance visitor enjoyment with animal welfare [3], though this can be challenging when their goals are in conflict. For example, the current research suggests that visitors are seeking connections with animals, but these perceived connections by visitors could be harmful to animals. This research demonstrates the importance of allowing visitors to connect with animals in a controlled way which does not reduce welfare. Supervised feeding experiences [14] or animal–visitor enrichment activity [72] could be a way to facilitate this. The current research illuminates different aspects of negative visitor behaviour and highlights that negative behaviour expression varies amongst different species and enclosure types. Understanding this could help zoos focus on creating signage or stationing staff or volunteers at the appropriate exhibits. Additionally, the research shows which visitors are most likely to engage in unwanted behaviours, which will enable zoos to use interpretation to focus on those visitors. More detailed research is needed on the impact of negative actions on animal behaviour and also in different regions of the world where religious or cultural differences may impact behaviour. Ultimately, ensuring the welfare of captive animals is the zoos’ responsibility. By understanding their visitors and the behaviours they engage in, zoos can enhance their animals’ welfare.

## 5. Conclusions

The aim of this research was to investigate the prevalence and type of negative behaviour at four different zoos and to consider which variables contributed to undesirable visitor actions. The results showed that negative visitor behaviours were not uncommon and that, across all institutions, banging, shouting and climbing barriers were the most commonly observed negative behaviours. Safari Ramat Gan experienced the most negative behaviours, while Mandai Wildlife Group had the least. The most ‘harassed’ species were those considered charismatic. Children, men and family groups were the most likely to engage in negative behaviours, and this was more likely as visitor numbers increased. These unwanted actions were not usually accompanied by conversational comments, but those that were overheard tended to be managerial. Negative behaviours generally occurred when animals were active and in close proximity to visitors. There was little association between staff perception of visitor behaviour and their actual behaviour. Surprisingly, enclosure type did not affect negative visitor behaviour. It is important for zoos to understand negative visitor behaviours so that they may begin to mitigate them.

## Figures and Tables

**Table 1:** (**A**–**D**) Details of each zoo. (**A**) Fota Wildlife Park, (**B**) Mandai Wildlife Group, (**C**) National Zoological Garden and (**D**) Safari Ramat Gan and the animal enclosures that were included in this study. Visitor range and negative behaviours represent all observation sessions.

A. Institution	Fota Wildlife Park, Carrigtwohill, County Cork, Ireland (FWP)									
Annual visitor attendance †	Pre-COVID: 450,000					During the COVID-19 pandemic: 385,000				
Data collected	July–August 2020									
Weather	Data were collected during the Irish summertime. It was typically mild and dry with temperatures ranging from 15 °C to 24 °C with a mean daily temperature of 21 °C during data collection.									
	**Species**	**No. of animals in the enclosure**	**Mixed species**	**Type of enclosure**	**Enclosure dimensions**	**Signage present at enclosure**	**Negative behaviours**	**Visitor range and mean**	**Staff perception**	**Total neg. behaviours observed**
1.	Sumatran tiger (*Panthera tigris sumatrae*)	4	No	Walk through	6737 m^2^	No	Banging, shouting, climbing over barrier, throwing	135–259 X = 178	1	26
2.	Humboldt penguins (*Spheniscus humboldti*)	26	No	Naturalistic	2181 m^2^	Yes	Climbing over barrier, touching, feeding, banging, shouting, throwing	102–169 X = 122	3	29
3.	White-faced saki (*Pithecia pithecia*)	4	No	Traditional	33 m^2^	Yes	Banging, shouting	34–67; X = 45	3	28
4.	Siamang gibbon (*Symphalangus syndactylus*)	4	No	Naturalistic	555 m^2^	No	Throwing, shouting, banging	40–104; X = 76	2	6
5.	Meerkat (*Suricatta suricatta*)	4	Yes	Naturalistic	700 m^2^	No	Banging, shouting	38–63; X = 48	2	11
6.	Cheetah (*Acinonyx jubatus*)	10 ^	No	Naturalistic	1112 m^2^	Yes	Climbing over barrier, touching, feeding, banging, shouting, throwing	60–177; X = 115	2	11
**B. Institution**	**Mandai Wildlife Group, Singapore (MWG)**									
Annual visitor attendance †	Pre-COVID: 4.5–5 million visitors					During the COVID-19 pandemic: approximately 4.7 million				
Data collected	August 2020–July 2021									
Weather	Weather was consistently hot and generally humid with some rain with temperatures ranging from 30 °C to 32 °C.									
	**Species**	**No. of animals in the enclosure**	**Mixed species**	**Type of enclosure**	**Enclosure dimensions**	**Signage present at enclosure**	**Negative behaviours**	**Visitor range and mean**	**Staff perception**	**Total neg. behaviours observed**
1.	Bengal Tiger (*Panthera tigris tigris*)	2	No	Naturalistic	1232.5 m^2^	Yes	Climbing barrier, feeding, throwing	47–163; X = 109	4	7
2.	African Penguin (*Spheniscus demersus*)	12	No	Naturalistic	35 m^2^ **	Yes	Banging, throwing, feeding, flash-photography, climbing barrier	10–122; X = 57	4	2
3.	Proboscis Monkey (*Nasalis larvatus*)	6	No	Naturalistic	96 m^2^	No	Banging, flash photography	4–50; X = 24	2	0
4.	Bornean Orangutan (*Pongo pygmaeus*)	4	Yes	Naturalistic	1400 m^2^	Yes	Feeding, throwing, climbing barrier	12–137 X = 79	2	0
5.	Meerkat (*Suricata suricatta*)	3	No	Naturalistic	12 m^2^	No	Feeding, throwing, climbing barrier	8–23; X = 14	1	0
6.	King cobra (*Ophiophagus hannah*)	1	No	Naturalistic	7.5 m^2^	No	Banging, flash photography	2–22 X = 16	3	15
7.	Giant panda (*Ailuropoda melanoleuca*)	1	No	Walk through	2800 m^2^	Yes	Banging, feeding, throwing, climbing barrier, shouting	40–149 X = 102	5	33
**C. Institution**	**National Zoological Garden, South Africa (NZG)**									
Annual visitor attendance †	Pre-COVID: 428,163					During the COVID-19 pandemic: 121,347				
Data collected	November 2020–January 2021									
Weather	Data were collected during the South African summertime. It was typically hot and dry with temperatures ranging from 26 °C to 32 °C with a mean daily temperature of 29 °C.									
	**Species**	**No. of animals in the enclosure**	**Mixed species**	**Type of enclosure**	**Enclosure dimensions**	**Signage present at enclosure**	**Negative behaviours**	**Visitor range and mean**	**Staff perception**	**Total neg. behaviours observed**
1.	Lion (*Panthera leo*)	3	No	Naturalistic	2500 m^2^	No	Banging, shouting, climbing barrier, throwing	25–41; X = 35	2	24
2.	African penguin (*Spheniscus demersus*)	28	No	Traditional	367 m^2^	No	Banging, shouting, touching, feeding	6–21; X = 10	2	24
3.	Ring-tailed lemur (*Lemur catta*)	12	No	Traditional	60 m^2^	No	Banging, shouting	7–21; X = 13	2	28
4.	Chimpanzee (*Pan troglodytes*)	2	No	Naturalistic	1000 m^2^	Yes	Banging, shouting	2–8; X = 5	3	5
5.	Meerkat (*Suricatta suricatta*)	7	No	Traditional	16 m^2^	No	Banging, shouting, climbing barrier, touching, feeding	2–12; X = 7	2	8
6.	Komodo dragon (*Varanus komodoensis*)	1	No	Traditional	100 m^2^	No	Banging, shouting	0–7; X = 3	1	5
**D. Institution**	**Safari Zoological Center Ramat-Gan, Israel (SRG)**									
Annual visitor attendance †	Pre-COVID: 705,000					During the COVID-19 pandemic: 514,000				
Data collected	August 2021									
Weather	Weather was generally hot with temperatures in the range of 28–32 °C, with a mean daily temperature of 31 °C.									
	**Species**	**No. of animals in the enclosure**	**Mixed species**	**Type of enclosure**	**Enclosure dimensions**	**Signage present at enclosure**	**Negative behaviours**	**Visitor range and mean**	**Staff perception**	**Total neg. behaviours observed**
1.	African Penguin *(Spheniscus demersus*)	20	No	Traditional	156 m^2^	Yes	Feeding, throwing, touching, banging, shouting	11–104; X = 37 * * based on 8 days	5	12
2.	Lion-tailed macaque (*Macaca silenus*)	12	No	Traditional	200 m^2^	Yes	Fence jumping, feeding, shoving objects into enclosure, and throwing	11–48; X = 33 * * based on 7 days	5	21 * * 9 days of observation
3.	Western lowland gorilla (*Gorilla gorilla*) Enclosure with glass	6	No	Traditional	980 m^2^	Yes	Throwing, banging, shouting, urinating on glass	13–84; X = 46 * * based on 8 days	5	34
4.	Meerkat (*Suricata suricatta*)	7	No	Naturalistic	129 m^2^	Yes	Fence jumping, throwing, shoving objects, feeding, shouting, banging	24–115; X = 52 * * based on 8 days	4	42
5.	Nile crocodile (*Crocodylus niloticus*)	4	No	Traditional	700 m^2^	Yes	Fence jumping, spitting and throwing	17–112; X = 44 * * based on 8 days	5	14
6.	African spurred tortoise (*Geochelone sulcata*)	6	No	Traditional	90 m^2^	Yes	Fence jumping, throwing, touching	14–101; X = 48 * * 6 days	3	10 * * 9 days of observation
7.	Petting zoo goats and sheep (*Ovis aries* spp.) and (*Capra hircus* spp.)	13	Yes	Semi-free ranging	890 m^2^	Yes	Feeding, shouting, grabbing, mounting animals or barriers	35–116; X = 68 * * 9 days	5	19

^ Note: Three adjacent enclosures were simultaneously observed for this species. † These numbers are approximate and include school groups and scheduled tours. ** These are the dimensions of the indoor penguin exhibit, where penguins were located when observations occurred, though visitor viewing is outdoors. * Some of the data were missing.

**Table 3:** Remaining explanatory variables after backwards selection, estimate, standard error, *p*-values and degrees of freedom information for the model.

Variables Remaining in the Model	Estimate	Standard Error	*p*-Value	DF
Zoo *	−0.310	0.386	*p* < 0.001	3
Species *	0.312	0.356	*p* < 0.001	6
Number of visitors	0.017	0.004	*p* < 0.001	1
Zoo * Species *	−4.021	0.601	*p* < 0.001	12

* Categorical values are averaged.

**Table 4:** The mean rate of negative behaviours observed per observation session in total and at FWP, MWG, NZG and SRG for each exhibit observed. NA indicates that the species did not occur at the specific institution, while 0.0 indicates that no negative behaviours were observed.

	Total	FWP	MWG	NZG	SRG
**Big cat**	1.9	2.6	0.7	2.4	NA
**Penguin**	1.7	2.9	0.2	2.4	1.2
**Meerkat**	1.5	1.1	0.0	0.8	4.2
**Primate**	2.0	2.8	0.0	2.8	2.3
**Ape**	1.1	0.6	0.0	0.5	3.4
**Reptile**	1.1	NA	1.5	0.5	1.3
**Charismatic species**	2.1	1.1	3.3	NA	1.9

**Table 5:** Fisher’s Exact and the Chi-Square test and Cramer’s V comparing observed negative behaviours to age, gender, proximity, activity and zoo.

			Bang	Shout	Feed	Touch	Throw	Chase/Kick	Climb Fence/Enter Enclosure	Other
Variables	Categories	Statistics *								
Age **	Adult Child	***p* < 0.001** **φ = 0.360**	n = 45 n = 128	n = 13 n = 99	n = 15 n = 7	n = 14 n = 9	n = 4 n = 11	n = 0 n = 4	n = 13 n = 42	n = 5 n = 3
Gender	Male Female	*p* = 0.876 φ = 0.086	n = 115 n = 58	n = 73 n = 39	n = 13 n = 9	n = 17 n = 6	n = 9 n = 6	n = 3 n = 1	n = 33 n = 22	n = 4 n = 4
Proximity	Far Near	***p* < 0.001** **φ = 0.341**	n = 19 n = 154	n = 8 n = 104	n = 6 n = 16	n = 0 n = 23	n = 4 n = 11	n = 3 n = 1	n = 5 n = 50	n = 5 n = 3
Activity level	Inactive Active	***p* < 0.001** **φ = 0.298**	n = 18 n = 155	n = 11 n = 101	n = 8 n = 14	n = 2 n = 21	n = 4 n = 11	n = 3 n = 1	n = 7 n = 48	n = 4 n = 4
Zoo **	FWP MWG NZG SRG	***p* < 0.001** **φ = 0.365**	n = 64 n = 17 n = 39 n = 53	n = 11 n = 26 n = 54 n = 21	n = 1 n = 0 n = 0 n = 21	n = 6 n = 0 n = 0 n = 17	n = 9 n = 0 n = 0 n = 6	n = 0 n = 0 n = 0 n = 4	n = 19 n = 13 n = 1 n = 22	n = 1 n = 0 n = 0 n = 7

* Cramer’s V (φ) is a measure of association where >0 none, >0.05 weak, >0.10 moderate, >0.15 strong and >0.25 very strong [51]; ** The Chi-square test was applied to these data; significant results are highlighted in bold text; α = 0.003 after the Bonferroni correction.

## Data Availability

Data are available upon request from the corresponding author.

## References

[1] Sherwen S.L., Hemsworth P.H. (2019). The visitor effect on zoo animals: Implications and opportunities for zoo animal welfare. Animals.

[2] Hosey G. (2008). A preliminary model of human–animal relationships in the zoo. Appl. Anim. Behav. Sci..

[3] Fernandez E.J., Tamborski M.A., Pickens S.R., Timberlake W. (2009). Animal–visitor interactions in the modern zoo: Conflicts and interventions. Appl. Anim. Behav. Sci..

[4] Altman J.D. (1998). Animal activity and visitor learning at the zoo. Anthrozoös.

[5] Finlay T., James L.R., Maple T.L. (1988). People’s perceptions of animals: The influence of zoo environment. Environ. Behav..

[6] Moss A., Esson M. (2010). Visitor interest in zoo animals and the implications for collection planning and zoo education programmes. Zoo Biol..

[7] Skibins J.C., Powell R.B. (2013). Conservation caring: Measuring the influence of zoo visitors’ connection to wildlife on pro-conservation behaviors. Zoo Biol..

[8] Luebke J.F., Watters J.V., Packer J., Miller L.J., Powell D.M. (2016). Zoo visitors’ affective responses to observing animal behaviors. Visit. Stud..

[9] Collins C., Corkery I., McKeown S., McSweeney L., Flannery K., Kennedy D., O’Riordan R. (2020). An educational intervention maximizes children’s learning during a zoo or aquarium visit. J. Environ. Educ..

[10] Godinez A.M., Fernandez E.J. (2019). What is the zoo experience? How zoos impact a visitor’s behaviors, perceptions, and conservation efforts. Front. Psychol..

[11] Hosey G.R. (2005). How does the zoo environment affect the behaviour of captive primates?. Appl. Anim. Behav. Sci..

[12] Jamieson D., Singer P. (1985). Against Zoos. In Defense of Animals.

[13] D’Cruze N., Khan S., Carder G., Megson D., Coulthard E., Norrey J., Groves G. (2019). A global review of animal–visitor interactions in modern zoos and aquariums and their implications for wild animal welfare. Animals.

[14] Fernandez E.J., Upchurch B., Hawkes N.C. (2021). Public feeding interactions as enrichment for three zoo-housed elephants. Animals.

[15] de Mori B., Ferrante L., Florio D., Macchi E., Pollastri I., Normando S. (2019). A protocol for the ethical assessment of wild animal–visitor interactions (AVIP) evaluating animal welfare, education, and conservation outcomes. Animals.

[16] Spooner S.L., Farnworth M.J., Ward S.J., Whitehouse-Tedd K.M. (2021). Conservation Education: Are Zoo Animals Effective Ambassadors and Is There Any Cost to Their Welfare?. J. Zool. Bot. Gard..

[17] Clayton S., Fraser J., Saunders C.D. (2009). Zoo experiences: Conversations, connections, and concern for animals. Zoo Biol..

[18] Learmonth M.J., Sherwen S., Hemsworth P.H. (2021). Assessing preferences of two zoo-housed Aldabran giant tortoises (*Aldabrachelys gigantea*) for three stimuli using a novel preference test. Zoo Biol..

[19] Hosey G.R. (2000). Zoo animals and their human audiences: What is the visitor effect?. Anim. Welf..

[20] Collins C.K., Quirke T., Overy L., Flannery K., O’Riordan R. (2016). The effect of the zoo setting on the behavioural diversity of captive gentoo penguins and the implications for their educational potential. J. Zoo Aquar. Res..

[21] Sherwen S.L., Magrath M.J., Butler K.L., Phillips C.J., Hemsworth P.H. (2014). A multi-enclosure study investigating the behavioural response of meerkats to zoo visitors. Appl. Anim. Behav. Sci..

[22] Carder G., Semple S. (2008). Visitor effects on anxiety in two captive groups of western lowland gorillas. Appl. Anim. Behav. Sci..

[23] Stoinski T.S., Jaicks H.F., Drayton L.A. (2012). Visitor effects on the behaviour of captive western lowland gorillas: The importance of individual differences in examining welfare. Zoo Biol..

[24] Tetley C.L., O’Hara S.J. (2012). Ratings of animal personality as a tool for improving the breeding, management and welfare of zoo mammals. Anim. Welf. UFAW J..

[25] Blaney E., Wells D. (2004). The influence of a camouflage net barrier on the behaviour, welfare and public perceptions of zoo-housed gorillas. Anim. Welf..

[26] Morgan K.N., Tromborg C.T. (2007). Sources of stress in captivity. Appl. Anim. Behav. Sci..

[27] Chiew S.J., Butler K.L., Sherwen S.L., Coleman G.J., Fanson K.V., Hemsworth P.H. (2019). Effects of regulating visitor viewing proximity and the intensity of visitor behaviour on little penguin (*Eudyptula minor*) behaviour and welfare. Animals.

[28] Collins C., Quirke T., McKeown S., Flannery K., Kennedy D., O’Riordan R. (2019). Zoological education: Can it change behaviour?. Appl. Anim. Behav. Sci..

[29] Mitchell G., Tromborg C.T., Kaufman J., Bargabus S., Simoni R., Geissler V. (1992). More on the ‘influence’ of zoo visitors on the behaviour of captive primates. Appl. Anim. Behav. Sci..

[30] Quadros S., Goulart V.D., Passos L., Vecci M.A., Young R.J. (2014). Zoo visitor effect on mammal behaviour: Does noise matter?. Appl. Anim. Behav. Sci..

[31] Moss A.G., Pavitt B. (2019). Assessing the effect of zoo exhibit design on visitor engagement and attitudes towards conservation. J. Zoo Aquar. Res..

[32] Ross S.R., Lukas K.E. (2005). Zoo visitor behavior at an African ape exhibit. Visit. Stud. Today.

[33] Collins C.K., McKeown S., O’Riordan R. (2023). A comprehensive investigation of negative visitor behaviour in the zoo setting and captive animals’ behavioural response. Heliyon.

[34] Kratochvil H., Schwammer H. (1997). Reducing acoustic disturbances by aquarium visitors. Zoo Biol..

[35] Tay C., McWhorter T.J., Xie S., Mohd Nasir T.S.B., Reh B., Fernandez E.J. (2023). A comparison of staff presence and signage on zoo visitor behavior. Zoo Biol..

[36] Ward S.J., Williams E., Groves G., Marsh S., Morgan D. (2020). Using zoo welfare assessments to identify common issues in developing country zoos. Animals.

[37] Tishler C., Assaraf O.B.Z., Fried M.N. (2020). How Do Visitors from Different Cultural Backgrounds Perceive the Messages Conveyed to Them by Their Local Zoo?. Interdiscip. J. Environ. Sci. Educ..

[38] Spannring R. (2017). Animals in environmental education research. Environ. Educ. Res..

[39] Wallace R. (2021). Front pages are for the charismatic: The case of the cute giant panda. Communicating Endangered Species.

[40] Harley J.J., Rowden L.J., Clifforde L.M., Power A., Stanley C.R. (2022). Preliminary investigation of the effects of a concert on the behavior of zoo animals. Zoo Biol..

[41] Martin P., Bateson P. (2007). Measuring Behaviour: An Introductory Guide.

[42] Moss A., Esson M., Francis D. (2010). Evaluation of a third-generation zoo exhibit in relation to visitor behavior and interpretation use. Editor. Assist..

[43] Moss A., Francis D., Esson M. (2008). The relationship between viewing area size and visitor behavior in an immersive Asian elephant exhibit. Visit. Stud..

[44] Mun J.S.C., Kabilan B., Alagappasamy S., Guha B. (2013). Benefits of Naturalistic Free-Ranging Primate Displays and Implications for Increased Human–Primate Interactions. Anthrozoös.

[45] Collins C., Corkery I., Haigh A., McKeown S., Quirke T., O’Riordan R. (2017). The effects of environmental and visitor variables on the behavior of free-ranging ring-tailed lemurs (*Lemur catta*) in captivity. Zoo Biol..

[46] Tunnicliffe S.D. (2005). Do Your Visitors Talk about Your Exhibits? What Do They Say? Presentation Given at Visitor Studies Day: Victoria and Albert Museum, London, UK. http://www.leeds.ac.uk/educol/documents/168630.htm.

[47] Collins C., McKeown S., McSweeney L., Flannery K., Kennedy D., O’Riordan R. (2021). Children’s conversations reveal in-depth learning at the Zoo. Anthrozoös.

[48] Altmann J. (1974). Observational study of behavior: Sampling methods. Behaviour.

[49] Denham B.E. (2017). Poisson and negative binomial regression. Categorical Statistics for Communication Research.

[50] IBM Corp (2021). IBM SPSS Statistics for Windows, Version 28.

[51] Akoglu H. (2018). User’s guide to correlation coefficients. Turk. J. Emerg. Med..

[52] Tunnicliffe S.D., Lucas A.M., Osborne J. (1997). School visits to zoos and museums: A missed educational opportunity?. Int. J. Sci. Educ..

[53] Clayton S., Luebke J., Saunders C., Matiasek J., Grajal A. (2014). Connecting to nature at the zoo: Implications for responding to climate change. Environ. Educ. Res..

[54] Mooney A., Conde D.A., Healy K., Buckley Y.M. (2020). A system wide approach to managing zoo collections for visitor attendance and in situ conservation. Nat. Commun..

[55] Albert C., Luque G.M., Courchamp F. (2018). The twenty most charismatic species. PLoS ONE.

[56] Nash S., Paul R. (2022). The behavioural biology of captive reptiles. The Behavioural Biology of Zoo Animals.

[57] Howell T.J., McLeod E.M., Coleman G.J. (2019). When zoo visitors “connect” with a zoo animal, what does that mean?. Zoo Biol..

[58] Hosey G., Melfi V., Ward S.J. (2020). Problematic animals in the zoo: The issue of charismatic megafauna. Probl. Wildl. II New Conserv. Manag. Chall. Hum. Wildl. Interact..

[59] Myers O.E., Saunders C.D., Birjulin A.A. (2004). Emotional dimensions of watching zoo animals: An experience sampling study building on insights from psychology. Curator Mus. J..

[60] Skibins J.C., Dunstan E., Pahlow K. (2017). Exploring the influence of charismatic characteristics on flagship outcomes in zoo visitors. Hum. Dimens. Wildl..

[61] Farrand A., Hosey G., Buchanan-Smith H.M. (2014). The visitor effect in petting zoo-housed animals: Aversive or enriching?. Appl. Anim. Behav. Sci..

[62] Caro T.M. (2010). Conservation by Proxy: Indicator, Umbrella, Keystone, Flagship, and Other Surrogate Species.

[63] Cohen E. (2010). Panda and elephant–contesting animal icons in Thai tourism. J. Tour. Cult. Change.

[64] Price E.C., Ashmore L.A., McGivern A.M. (1994). Reactions of zoo visitors to free-ranging monkeys. Zoo Biol..

[65] Chiew S.J., Hemsworth P.H., Melfi V., Sherwen S.L., Burns A., Coleman G.J. (2021). Visitor attitudes toward little penguins (*Eudyptula minor*) at two Australian zoos. Front. Psychol..

[66] Ajzen I. (1985). From intentions to actions: A Theory of Planned Behavior. Action Control.

[67] Ajzen I. (1991). The theory of planned behavior. Organ. Behav. Hum. Decis. Process..

[68] Biel A., Thøgersen J. (2007). Activation of social norms in social dilemmas: A review of the evidence and reflections on the implications for environmental behaviour. J. Econ. Psychol..

[69] Terrier L., Marfaing B. (2015). Using social norms and commitment to promote pro-environmental behavior among hotel guests. J. Environ. Psychol..

[70] Jensen E. (2014). Evaluating children’s conservation biology learning at the zoo. Conserv. Biol..

[71] Choo Y., Todd P.A., Li D. (2011). Visitor effects on zoo orangutans in two novel, naturalistic enclosures. Appl. Anim. Behav. Sci..

[72] Collins C.K., McKeown S., O’Riordan R. (2021). Does an Animal–Visitor Interactive Experience Drive Conservation Action?. J. Zool. Bot. Gard..

